# Early Posttransplant Lymphoproliferative Disorder and Cryptosporidiosis After Liver Transplantation

**DOI:** 10.1155/carm/5215414

**Published:** 2026-05-23

**Authors:** Atousa Hakamifard, Sara Abolghasemi, Behzad Hatami, Forogh Mangeli, Mahnaz Momenzadeh

**Affiliations:** ^1^ Cancer Prevention Research Center, Isfahan University of Medical Sciences, Isfahan, Iran, mui.ac.ir; ^2^ Infectious Diseases and Tropical Medicine Research Center, Shahid Beheshti University of Medical Sciences, Tehran, Iran, sbmu.ac.ir; ^3^ Gastroenterology and Liver Diseases Research Center, Research Institute for Gastroenterology and Liver Diseases, Shahid Beheshti University of Medical Sciences, Tehran, Iran, sbmu.ac.ir; ^4^ Clinical & Anatomical Pathologist, Taleghani Hospital Pathology Ward, Shahid Beheshti University of Medical Sciences, Tehran, Iran, sbmu.ac.ir; ^5^ Department of Clinical Pharmacy and Pharmacy Practice and Pharmaceutical Sciences Research Center, Isfahan University of Medical Sciences, Isfahan, Iran, mui.ac.ir

## Abstract

Posttransplant lymphoproliferative disorder (PTLD) is a rare yet potentially fatal complication following liver transplantation. This condition is usually associated with Epstein–Barr virus (EBV) infection and profound immunosuppression. In this report, a young woman is introduced who had undergone liver transplantation due to autoimmune hepatitis and developed persistent diarrhea 3 months after the transplantation. Simultaneously, *Cryptosporidium* infection was diagnosed as the cause of the diarrhea. However, due to the persistence of symptoms and lack of full recovery with initial treatment, further diagnostic evaluations were conducted. Tissue biopsy confirmed the presence of diffuse large B‐cell lymphoma (DLBCL) as an early‐onset PTLD. Following the diagnosis of PTLD, a reduction in the intensity of immunosuppression and initiation of specific treatment, including the administration of rituximab, were undertaken. Nevertheless, despite the therapeutic interventions, the patient died due to disease progression. This case underscores the necessity of considering PTLD as a differential diagnosis in liver transplant patients presenting with unusual symptoms such as persistent diarrhea—even in the presence of a concurrent opportunistic infection like *Cryptosporidium*. Timely diagnosis and treatment of PTLD are crucial to improving the prognosis of these patients.

## 1. Introduction

Posttransplant lymphoproliferative disorders (PTLD) are a heterogeneous group of abnormal lymphoid proliferations arising in the context of immunosuppression in transplant recipients [1]. These proliferations range from benign polyclonal hyperplasia to malignant monoclonal lymphoma and can occur at any time after transplantation [2]. The prevalence of PTLD in solid organ transplant recipients has been reported at approximately 1%–3%, although this rate varies depending on the type of transplanted organ and the patient population [2]. In general, the risk of PTLD is higher in heart, lung, and small intestine transplants due to the need for more intense immunosuppression, compared to liver and kidney transplants. In liver transplantation, the cumulative incidence of PTLD has been reported to be about 1% at 18 months and 4%–5% at 15 years posttransplantation [3]. Although PTLD is a rare complication, it holds significant clinical importance as it can be life‐threatening; various studies have reported PTLD‐related mortality rates ranging from approximately 25%–60% [4]. Epstein–Barr virus (EBV) infection is known as the cause of most PTLD cases. A primary EBV infection in an immunosuppressed individual leads to uncontrolled proliferation of EBV‐infected B lymphocytes; in a healthy immune state, this proliferation is inhibited by T cells, but in patients undergoing immunosuppressive therapy, this inhibition is disrupted, paving the way for a spectrum of lymphoid proliferations. For this reason, most early‐onset PTLDs (within the first year after transplantation) are associated with EBV and are B‐cell in nature [5]. In contrast, late‐onset PTLDs (occurring several years posttransplant) are more often EBV‐negative, monomorphic (monotypic), and more aggressive. Other risk factors for PTLD include recipient EBV‐negative and donor EBV‐positive status (i.e., lack of prior immunity in the recipient), younger age particularly in children, the intensity and type of immunosuppressive regimen (such as the use of T‐cell depleting antibodies or potent agents like tacrolimus), and the underlying disease that led to transplantation [6]. Reports have indicated that in liver transplant recipients, autoimmune hepatitis as the transplant indication and steroid treatment prior to transplantation are considered independent risk factors for PTLD [7]. PTLD can present with a wide range of clinical manifestations and may involve any organ or tissue. Gastrointestinal involvement has been reported in 23%–56% of PTLD patients and can manifest with symptoms such as iron deficiency anemia, growth failure (in children), gastrointestinal bleeding, perforation, intussusception, bowel obstruction, or diarrhea [1]. Gastrointestinal involvement—particularly ulcers or masses in the stomach, small intestine, and colon—is a relatively common presentation of PTLD and may appear with chronic diarrhea, abdominal pain, or bleeding. In transplant patients, the simultaneous presence of cryptosporidiosis can delay diagnosis or complicate the clinical picture [8]. The initial therapeutic approach to PTLD generally includes reducing the intensity of immunosuppression, although this carries the risk of graft rejection. In many cases, especially in B‐cell PTLDs associated with EBV, treatment with rituximab (a monoclonal anti‐CD20 antibody) alone or in combination with low‐dose chemotherapy may lead to improvement [9, 10]. However, about 30%–40% of patients—particularly those with high‐risk monomorphic or EBV‐negative lesions—require intensive systemic chemotherapy (such as lymphoma regimens) to control the disease. Despite therapeutic advancements, the prognosis of PTLD remains variable, and in cases with disseminated disease or severe systemic symptoms, it is poor [9]. In this case report, we present a case of early‐onset PTLD of the diffuse large B‐cell lymphoma (DLBCL) type in a young woman who received a liver transplant due to autoimmune hepatitis and presented with persistent diarrhea. This patient was simultaneously affected by gastrointestinal *Cryptosporidium* infection. Her disease course was accompanied by severe complications (fever, neutropenia, gastrointestinal bleeding, and renal failure), which, despite therapeutic interventions, ultimately led to her death. The coexistence of early‐onset PTLD in an EBV‐seropositive recipient with *Cryptosporidium* infection is a rare phenomenon that highlights the necessity of clinical vigilance, regular monitoring of EBV load, and aggressive tissue‐based evaluation in liver transplant patients with persistent gastrointestinal symptoms.

## 2. Case Presentation

A 24‐year‐old female patient, recipient of a cadaveric liver transplant due to autoimmune hepatitis, has been under treatment with CellCept, prednisolone, and tacrolimus, along with cotrimoxazole (for PCP prophylaxis). Three months after transplant, she presented with a 20‐day history of nausea, vomiting, and diarrhea. Due to the persistence of symptoms and lack of improvement, she was admitted under the gastroenterology service. The patient’s diarrhea was watery, occurring eight times a day, with no tenesmus. On examination, the abdomen was soft and nontender. She was afebrile. The patient did not have lymphadenopathy.

Initial vital signs upon admission were as follows: RR = 17, PR = 100, BP = 80/60, and T = 37°C. The patient was alert, with no other associated symptoms. She started IV fluid therapy.

Pretransplant patient’s laboratory tests were as follows: CMV Ab IgG = 21 (positive), HSV 1, 2 Ab IgG = 1.5 (negative), EBV Ab IgG = 15 (positive), VZV Ab IgG = 36 (positive), HBS Ag = 0.4 (negative), HBS Ab = 103 (positive), HBC Ab (total) = 1.5 (nonreactive), HCV Ab = 0.1 (negative), and HIV Ab = 0.1 (negative).

Laboratory tests were ordered, and given the history of persistent diarrhea in an immunocompromised patient, a stool sample was sent for the following evaluations: Stool culture and analysis, *Giardia*, *Cryptosporidium*, *Microsporidium*, *Isospora*, and *Clostridium difficile* toxins A and B. Serum quantitative CMV PCR was requested. A quantitative EBV viral load was also sent for monitoring. Given the recent transplant and diarrhea history, oral vancomycin 250 mg every 6 h was initiated empirically for *Clostridium difficile* pending test results.

In the stool analysis, the diarrhea was classified as noninflammatory. The stool stain for *Cryptosporidium* was positive, and all the other tests were negative. Since nitazoxanide was not available in our region, paromomycin capsules at 500 mg every 6 h were prescribed. In addition, the doses of the patient’s immunosuppressive medications were reduced as much as possible. As diarrhea persisted and new symptoms of abdominal pain and vomiting appeared, an abdominal and pelvic CT scan with and without contrast was ordered.

**FIGURE 1:**
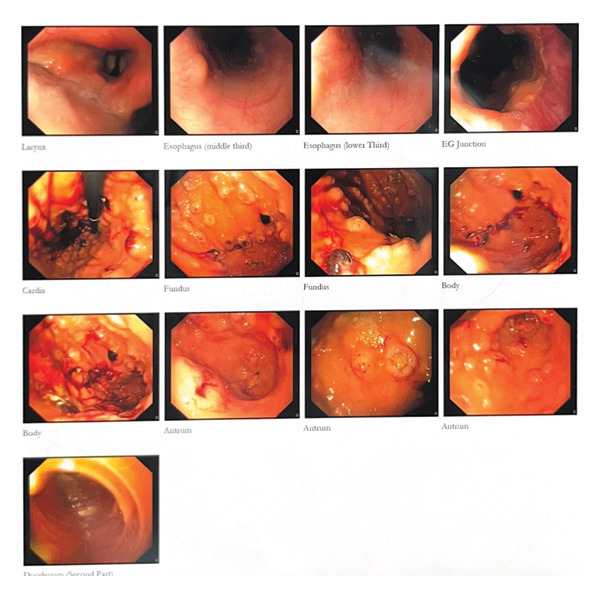
Stomach: Severe diffuse gastritis with numerous polypoid lesions with central ulcer (clean‐based, adherent clot). Multiple biopsies were obtained. Duodenum: duodenitis. Up to D3 was examined. Multiple polypoid lesions with central ulcers and oozing blood were seen in D2, involving the major and minor papillae.

**FIGURE 2:**
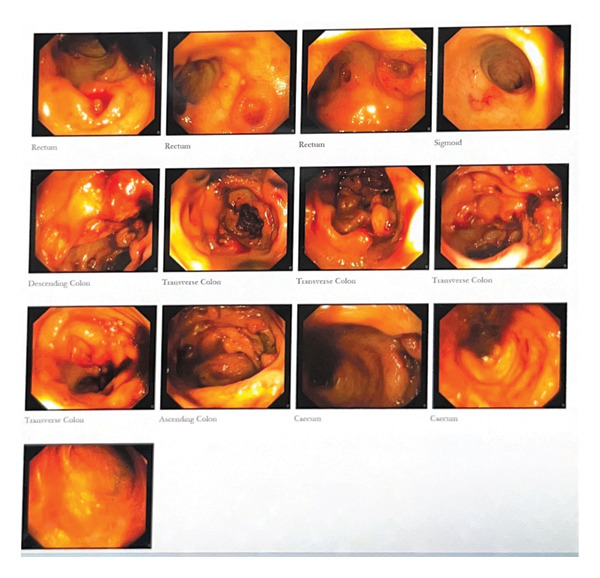
Numerous polypoid lesions with central ulcer were seen throughout the colon from the rectum up to the cecum. Multiple biopsies were taken. The mucosa between lesions appeared normal. T. ileum was normal.

Given the continued noninflammatory diarrhea, some episodes of mild bloody diarrhea, and liver and spleen lesions seen on CT, the patient became a candidate for upper endoscopy and colonoscopy. The report descriptions are shown in Figures 1 and 2.

**FIGURE 3:**
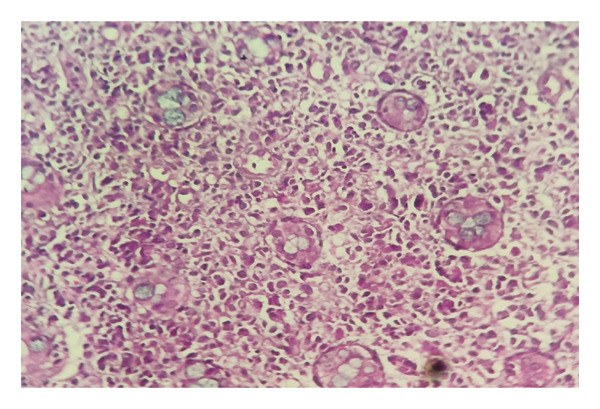
Hematoxylin & eosin (HE) staining, magnification, 10x. Neoplastic process composed of atypical large cells.

**FIGURE 4:**
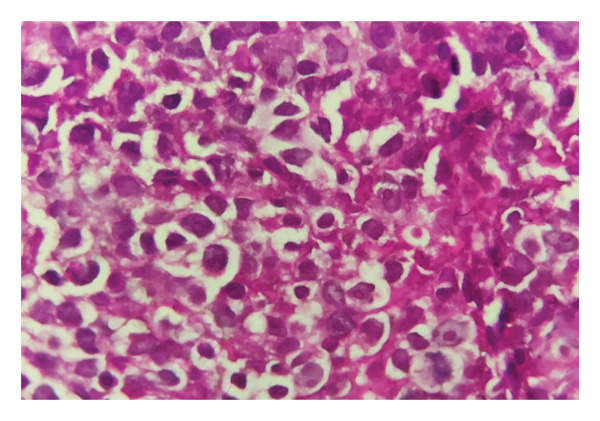
Hematoxylin and eosin (HE) staining, magnification, 40x. Diffuse infiltration of atypical large noncleaved lymphoid cells with oval, irregular vesicular nuclei, prominent nucleoli, and scant cytoplasm.

**FIGURE 5:**
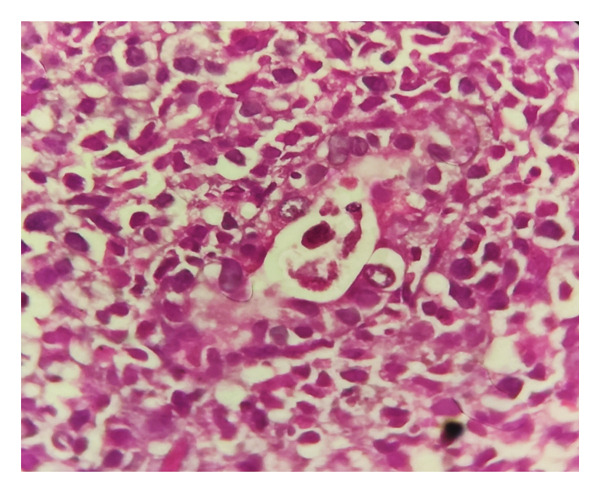
Hematoxylin and eosin (HE) staining, magnification, 40×. Lymphoepithelial lesion with infiltration of gastric glands by atypical lymphoid cells.

**FIGURE 6:**
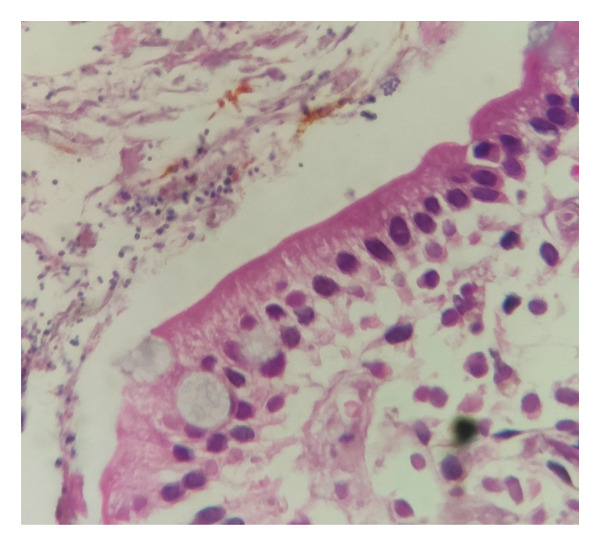
Hematoxylin & eosin (HE) staining, magnification, 100x. Numerous basophilic spherical extracellular organisms were found in the apex of enterocytes.

Histopathological examination of the gastric and colonic mucosa showed a neoplasm composed of atypical large noncleaved lymphoid cells with oval, irregular vesicular nuclei, prominent nucleoli, scant cytoplasm (Figures 3 and 4), and prominent areas of lymphoepithelial lesions (Figure 5). Microscopic findings of duodenal mucosa revealed numerous non–acid‐fast basophilic spherical extracellular bodies in surface epithelium with positive reaction in Giemsa staining (Figure 6). Pathology reports confirmed a diagnosis of PTLD with *Cryptosporidium* infection, and the sample was sent for IHC which was consistent with DLBCL.

Based on the endoscopic and colonoscopic findings, the liver and spleen lesions, and the reported serum EBV viral load of 777,034 copies/mL, CellCept was discontinued, and other immunosuppressive drugs were tapered as much as possible. CellCept was discontinued, and other immunosuppressive drugs were tapered as much as possible. Besides, lumbar puncture and bone marrow biopsy were performed. Both of which were normal. A PET‐CT was not performed for the patient. The patient’s bloody diarrhea was episodic and mild.

Following the DLBCL diagnosis, the patient was transferred from gastroenterology to the hematology‐oncology service. She became a candidate for chemotherapy with the R‐MiniCHOP regimen. Before initiation, the following were checked and came back negative: HBsAg, total HBc‐Ab, and IGRA.

Chemotherapy with the R‐MiniCHOP regimen was started. During hospitalization, 1 week after chemotherapy, the patient developed fever, thrombocytopenia, neutropenia, worsening gastrointestinal bleeding, and increased abdominal pain. Vital signs at the time were as follows: PR = 120, RR = 18, BP = 100/70, T = 38.4°C. Besides the platelet transfusions, the patient started on the following antibiotics: meropenem, vancomycin, and caspofungin. Besides, she received IV fluids with cardiac and respiratory monitoring. An NG tube was placed.

In addition, the *Clostridium difficile* infection (CDI) treatment regimen was restarted using oral vancomycin 500 mg every 6 h and IV metronidazole 500 mg every 8 h, due to fever, neutropenia, worsening abdominal pain, and bloody diarrhea, pending exclusion of probable typhlitis. Blood cultures were reported to be negative. The patient was transferred to the ICU. CT scans of the sinuses, lungs, abdomen, and pelvis were performed. The CD stool test was reported to be negative. Given persistent severe abdominal pain and continued bleeding, a surgical consultation was requested. Surgery was not recommended. Due to metabolic acidosis, rising creatinine, and becoming anuric, the patient became a candidate for emergency hemodialysis. She underwent two sessions of hemodialysis. Ultimately, 5 days later, the patient suffered cardiopulmonary arrest and passed away.

## 3. Discussion

The presented patient was a case of early‐onset PTLD following liver transplantation, manifested as DLBCL and progressed rapidly toward death. As mentioned, early‐onset PTLDs commonly occur in EBV‐naïve individuals (those lacking prior immunity) who are exposed to EBV posttransplantation [2]. In fact, the greatest PTLD risk among donor/recipient combinations corresponds to the scenario where the donor is EBV‐positive and the recipient is EBV‐negative [2]. But in this patient, the donor and recipient EBV status were both positive.

Approximately 3 months posttransplantation, our patient developed a very high EBV viral load (over 7 × 10^5^ copies/mL), indicating its unchecked proliferation due to immunosuppression. Pathophysiologically, following primary infection, EBV remains latent in B lymphocytes and expresses only a limited number of proteins (non‐EBNA nuclear antigens and LMP membrane proteins), helping it evade the immune system [11]. In a healthy individual, cytotoxic T cells recognize these antigens and suppress the proliferation of EBV‐infected B cells. However, in a patient like ours, who was receiving high doses of calcineurin inhibitors (tacrolimus) and other immunosuppressive agents, the T‐cell response to EBV is severely impaired. As a result, EBV gains the opportunity to transform B lymphocytes and induce their uncontrolled proliferation.

Another important risk factor in this patient was the underlying autoimmune hepatitis and its related treatments. Studies have shown that patients undergoing liver transplantation due to autoimmune conditions are more susceptible to PTLD than others [7]. In a study of 431 liver transplant recipients, both pretransplant steroid use and autoimmune hepatitis as the underlying diagnosis independently increased the likelihood of PTLD [7]. Our patient had both of these factors: She had undergone corticosteroid therapy prior to transplantation to control autoimmune hepatitis, and her active autoimmune disorder had led to the need for transplantation. The presence of underlying immune dysregulation may itself indicate a defect in immune control, which could facilitate lymphocyte proliferation after transplantation [12]. Furthermore, such patients often require relatively higher doses of immunosuppression posttransplant (due to immune system activation) to prevent graft rejection. The intensity of immune suppression is a key factor in the development of PTLD [2]. Strong T‐cell inhibition, such as that induced by tacrolimus or anti‐T‐cell antibodies, significantly increases PTLD risk [13]. Conversely, some immunosuppressants, like mTOR inhibitors (sirolimus, everolimus), may even exhibit antiproliferative effects and reduce the risk of PTLD [14].

Our case is particularly notable in terms of its initial presentation, as it manifested with persistent diarrhea. Diarrhea is common in transplant recipients and may result from various causes, ranging from infections to drug side effects [15]. In this patient, it was initially reasonable to consider diarrhea due to an opportunistic infection. The diagnosis of cryptosporidiosis actually supported this approach, as *Cryptosporidium* is a well‐known cause of persistent watery diarrhea in patients with impaired cellular immunity (including those on cyclosporine/tacrolimus or with AIDS) [15]. The key point here is that the presence of a single diagnosis (*Cryptosporidium*) should not prevent further evaluation for other potential causes. In our patient, although antiparasitic treatment was initiated, the lack of improvement in diarrhea along with the emergence of new symptoms (abdominal pain, mild bleeding) served as red flags, suggesting that the disease process extended beyond a simple parasitic infection. Therefore, timely imaging and endoscopy led to the discovery of lymphoproliferative lesions. In fact, PTLD can mimic a wide range of clinical presentations, and its diagnosis often requires a high index of suspicion and invasive diagnostic procedures.

Several cases have been reported in which gastrointestinal PTLD initially presented with diarrhea, GI bleeding, or ulcerative lesions [2]. For instance, in a similar case report, early‐onset PTLD following liver transplantation initially manifested as chronic diarrhea and extensive colonic ulcers, which were detected on colonoscopy and confirmed via biopsy as DLBCL [2].

The coexistence of *Cryptosporidium* infection and PTLD in this patient is noteworthy. On the one hand, the parasite was responsible for the patient’s diarrhea and had likely been affecting her gastrointestinal system for some time prior to the development of PTLD. *Cryptosporidium* can follow a prolonged and severe course in transplant recipients and may even spread to the biliary tract, leading to sclerosing cholangitis [15]. In a study of 47 transplant patients with cryptosporidiosis, 9% had biliary or pulmonary involvement and 3 patients (6%) died from the infection [15]. In our patient as well, the hepatic lesions seen on CT might have been caused by biliary microabscesses or *Cryptosporidium*‐induced cholangiopathy, although lymphoma involvement seemed more likely. In any case, the presence of this parasite complicated the patient’s condition, as persistent diarrhea could lead to dehydration, malabsorption, and weakness, making her more vulnerable to receiving chemotherapy. On the other hand, treatment of *Cryptosporidium* requires a reduction in immunosuppression [16], while the newly diagnosed lymphoma necessitated the continuation of some degree of immunosuppression (to prevent graft rejection) along with the initiation of specific chemotherapy.

In this patient, the treatment team was able to strike a relative balance by discontinuing CellCept and reducing the dose of tacrolimus allowing the immune system to fight *Cryptosporidium* while minimizing the risk of acute transplant rejection. Moreover, it is worth noting that opportunistic infections and PTLD can mimic or aggravate each other. Therefore, physicians should be mindful of potential coinfections in such complex patients. In this case, the simultaneous detection of lymphoma and *Cryptosporidium* in the duodenal biopsy confirmed this co‐occurrence. Similar reports of PTLD coexisting with opportunistic infections such as cytomegalovirus, aspergillosis, and others exist, each requiring separate treatment and presenting the challenge of concurrent management [17].

The treatment of PTLD is staged and based on disease severity. The first step is reduction of immunosuppression (e.g., reducing calcineurin inhibitors by 50% and discontinuing antimetabolites) [18]. In cases of monomorphic, CD20‐positive PTLD, treatment begins with rituximab (anti‐CD20); this can be administered as monotherapy or in combination with chemotherapy [2]. If the response is inadequate, combination chemotherapy such as R‐CHOP (rituximab with cyclophosphamide, doxorubicin, vincristine, and prednisone) or its reduced‐intensity version (R‐mini‐CHOP) is used [18]. Clinical studies have shown that a sequential treatment strategy (starting with rituximab followed by CHOP) induces complete or partial responses in over 90% of patients [18]. In similar case reports, patients with chronic diarrhea after liver transplantation improved following a single course of rituximab monotherapy [2]. After a successful treatment response, retransplantation may be considered if necessary, typically after at least 1 year [2]. In our patient as well, due to her frail physical condition and concern over complications, R‐miniCHOP was selected. The decision to initiate low‐dose R‐miniCHOP was based on the patient’s overall clinical condition. Although the patient was young, he had multiple comorbidities and was clinically assessed as frail, rendering full‐dose standard R‐CHOP potentially unsafe. R‐miniCHOP is a dose attenuated chemotherapy regimen specifically recommended for patients with compromised functional status or increased treatment‐related risk. Therefore, this regimen was selected to balance treatment efficacy with tolerability and safety. The lack of a formal risk assessment was acknowledged as a limitation of this report. However, based on a comprehensive clinical evaluation, the patient was categorized as unfit and frail. According to current guidelines, risk and age assessment are recommended even in younger patients with lymphoma to guide treatment selection. This principle was applied in our case, and the decision to use R‐miniCHOP was made on the basis of the patient’s clinical condition and perceived treatment‐related risk.

Studies indicate that PTLD diagnosed within the first year posttransplant and those with monoclonal tumors are associated with poorer prognoses. Other factors, such as multiorgan involvement (more than one site), poor functional status of the patient, and high‐risk histologic types (e.g., T‐cell lymphoma or CNS involvement), are linked to reduced survival [11].

In our patient, several of these factors were present: diagnosis within 3 months posttransplant (very early), monomorphic DLBCL, multiorgan involvement (diffuse GI, liver, spleen), and a clinical condition that rapidly deteriorated. Sadly, even aggressive treatment could not reverse the course of the disease. This underscores the need for earlier preventive and diagnostic interventions to improve outcomes.

The outcome of PTLD is variable. In case reports, a good treatment response is often associated with recovery; for instance, in the study by Waknine and Estrahutin, the mentioned OLT patient’s symptoms resolved after four doses of rituximab [2]. However, mortality has also been reported in advanced cases. In a series of five pediatric liver transplant patients with gastrointestinal PTLD, three (60%) died despite chemotherapy [19]. Larger studies suggest that PTLD survival depends on various factors, including patient age and disease stage [20]. Overall, raising clinical awareness, regular EBV monitoring, and early biopsy‐based diagnosis may improve clinical outcomes [20].

In our case, had EBV DNA been checked 1 month posttransplant, the rising viral trend might have been detected earlier, possibly allowing for more timely intervention. Being prepared for multiple diagnoses in transplant patients is essential.

Future treatments may involve novel approaches such as EBV‐specific T cells or CAR‐T cell therapy [9]. Besides, the role of antiviral agents (like ganciclovir) as adjunctive treatment in EBV‐positive PTLD remains a subject of debate; although these drugs have no effect on the latent phase of the virus, they may help control viremia by reducing the production of free virus and the infection of new cells [21].

## 4. Conclusion

This report presents a case of very early‐onset PTLD of the DLBCL type in a young liver transplant recipient, who presented with gastrointestinal symptoms and concurrent *Cryptosporidium* infection. This case highlights the importance of broad diagnostic thinking when facing ambiguous symptoms in immunocompromised patients and emphasizes the need for vigilant monitoring of EBV and other infections after transplantation. Reasonable reduction of immunosuppression intensity, prompt diagnostic biopsies in suspicious cases, and initiation of appropriate treatment (such as rituximab and chemotherapy) are fundamental to PTLD management.

## Author Contributions

A.H. contributed to case selection, study design, and manuscript revision. S.A., B.H., and F.M. contributed to case selection and study design. M.M. contributed to database searches and extraction of clinical studies and patient paraclinical information and writing of the original draft.

## Funding

The authors have nothing to report.

## Disclosure

All authors wrote the original draft and have read and approved the final version of the manuscript.

## Consent

Informed consent was obtained from the participant.

Written informed consent was obtained from the patient for publication of this case report.

## Conflicts of Interest

The authors declare no conflicts of interest.

## Data Availability

The data that support the findings of this study are available from the corresponding author upon reasonable request.
